# Hearing: a pediatrician's approach

**DOI:** 10.1016/S1808-8694(15)31017-X

**Published:** 2015-10-19

**Authors:** Angela Maria Fontana Zocoli, Fabiana Coelho Riechel, Bianca Simone Zeigelboim, Jair Mendes Marques

**Affiliations:** a MS student in Communications Disorders - University of Tuiuti - Paraná State, Speech and Hearing Therapist.; b Postgraduate Student in Clinical Audiology - University of Tuiuti - Paraná State, Speech and Hearing Therapist.; c PhD in Human Communications Disorders - UNIFESP/Escola Paulista de Medicina, Professor - Course of Specialization in Clinical Audiology and Coordinator of the Master's Degree Program in Communications Disorders - University of Tuiuti - Paraná, Speech and Hearing Therapist.; d PhD in Geodesic Sciences - Federal University of Paraná and Professor of the Postgraduate Program in Communications Disorders - University of Tuiuti - Paraná.

**Keywords:** Hearing Disorders, Pediatrics, Children

## Abstract

Hearing plays a fundamental role in a child's global development; however, some professionals do not realize how much they may contribute to mitigate the sequelae caused by hearing impairment.

**Aim:**

to collect data on pediatricians’ approaches in a city in the country side of Santa Catarina State, regarding the early detection of hearing impairment and identify the methodology utilized for its diagnosis.

**Study Design:**

Historical Cohort with Cross-Sectional Cohort.

**Materials and Methods:**

Analysis of questionnaires with nine multiple choice questions and ten open questions.

**Results:**

62% reported that their training in hearing disorders happened during their medical course; high risk patients are referred to the otorhinolaryngologist (92%); 83% said they are aware of the evaluation techniques; 55% stated they were not aware of the different types of hearing loss; only 25% reported they knew about the levels of hearing loss and 42% of the interviewees believe a child may have fruitful use of a hearing aid before six months of age.

**Conclusion:**

There is the need of more information about the importance of early diagnosis, as well as the methods used for this end.

## INTRODUCTION

At birth, human beings have only the so called reflex-type hearing, which is inhibited when the learning process starts, and new responses to sound crop up depending on the hearing experiences^1^. A baby's first year of life is full of learning. Though auditory feedback we receive the basic concepts necessary for language buildup, organized in different neuropsychological, organic and emotional processes, thus allowing symbolic learning^2^. Hearing, as a special sense itself, comes later, for the child stores up all sorts of information without sorting. Sounds start to have a real meaning when the process of learning to hear sets in, and such process may be influenced by the environment^1^.

It is known that language alteration causes may vary. An investigation method, seeking the precise diagnosis, may guide the health care professional in the choice of treatment for each case^3^.

The close relation between hearing and language acquisition is peculiar to the human being. The poorer the speech stimulation, smaller and less effective will be the language acquisition process^4^. This relationship between hearing and language development makes this early diagnosis of hearing impairment of the utmost importance^1^.

To prevent hearing loss is a means to protect and prevent the child from suffering the deleterious effects caused by the lack of auditory stimulation on language as a function^1^.

About 50% of hearing losses could be avoided or at lest have its sequelae reduced if we could have early measures that would identify, diagnose and rehabilitate hearing impaired children^5^.

Hearing impairment prevalence is twenty fold higher than other diseases such as phenylketonuria or hypothyroidism and its identification requires a cost that is ten fold lower than that for these diseases^6^.

The American Academy of Pediatrics reveals that permanent hearing impairment affects from one to three babies in every 1000 that are born and followed in regular nurseries; and from two to four babies among some 1000 which are born and followed up in neonatal ICUs. It also affects pre-school aged children, because of diseases such as meningitis and secretory otitis media acquired before the first twelve months of life^7^.

If one considers only the past history of exposure to risk factors in order to make the diagnosis of hearing loss, only 50% of the children will be properly diagnosed^4^, ^5^, ^6^, ^11^, ^12^.

The pediatrician's participation is paramount for the success of early hearing impairment detection and intervention programs, however there is also this need to involve the whole team of nurses, the families and the community. Even with all the information we have about neonatal screening, many professionals are still not familiarized with such principles^8^.

The process of early detection of hearing loss must start in the nurseries, through neonatal hearing screening, for it is an efficient means of identifying mainly the children at risk. Notwithstanding, screening by itself does not bring about any further benefit to the child's health, it only identifies the problem. The correct thing to do is to, right after birth, identify children at risk, refer the ones under suspicion of hearing impairment and place them in therapeutic processes^9^. Hearing screening is only complete through identification, confirmation an rehabilitation for children with hearing impairment^10^.

Hearing loss treatment during childhood goes much beyond the medical setting, because the implications are deep and severe. Since this is the time in which the world is being introduced to the children and there is this possibility of them not being able to understand it, his/her communication process may be irreversibly affected^7^.

The Joint Committee on Infant Hearing (JCIH)^11^ together with the National Health Institute in the USA created the Universal Audiologic Screening Guide for all newborns, standardizing the stages and guidelines that should be followed in order to screen these children. These recommendations and guidelines aim at promoting the early diagnosis of hearing loss, taking into account the critical period of auditory development in the child, attempting to minimize the negative impact that hearing impairment has over the child's intellectual development, through early rehabilitation^6^.

The Brazilian Committee on Childhood Hearing Loss (CBPAI)^12^ recommends the implementation of Universal Neonatal hearing screening (TANU) for all children from birth until three months of age. In cases of confirmed hearing impairment there should be some educational intervention as of the sixth month of life. The American Academy of Pediatrics recommends the use of electrophysiological methods in neonatal hearing screening programs such as the ABR, and distortion product otoacoustic emmisions^5^.

ABR assesses neural integrity of auditory pathways all the way to the brain stem, through the recording of the electrophysiological waves generated in response to a sound stimulus and captured by surface electrodes placed on the head of the individual. DPOAE record the sound energy generated by the cochlea hair cells, in response to the sounds presented and recorded by a miniaturized microphone placed in the external auditory canal. These methods are fast, non-invasive and of easy application, both have pros and cons. It is not possible to perform screening through electrophysiological methods; it is, however, possible to investigate together with the risk factors the hearing behavior observation and study of the cochleo-eyelid reflex, bearing in mind that mild or unilateral losses may be missed by this method^12^.

The hearing apparatus is totally formed by birth, in order to have proper maturation of the auditory pathways at the brain stem it is necessary to establish the hearing loss diagnosis already in the first year of life^13^. The critical period for hearing (from birth to two years of age) corresponds to the time of greatest neuronal plasticity of the auditory pathway^6^. During this time, the central auditory system may be changed, depending on the quantity and quality of the external stimuli captured. The greater the number of stimuli, the greater is the number of connections between the inner ear and the cortex^6^. The child that does not receive proper language stimulation during the first two or three years of life will never have her/his language potential fully developed^1^.

For the hearing impaired, the degree of difficulty does not really matter, nor does the time when the loss occurred, the important thing is that a whole world of information has been blocked, and the physician, the educator, the family and the society should try, by all means available, to overcome hurdles in order to bring him/her closer to the community to live among peers, without prejudice^1^.

Since the pediatrician is the first professional to directly follow the child in the first months of life, the present study aims at gathering information about the approach this professional may have as far as hearing impairment is concerned, and his/her approach for an early diagnosis.

### Methods

This research involved pediatricians who work in public and private hospitals and/or private clinics, in a city in the country side of the State of Santa Catarina - Brazil.

The study was carried out both in a quantitative and qualitative fashion, from February to April of 2004, and the questionnaire applied by Barros, Galindo and Jacob^14^ in 2002, with modifications, was used for data collection, according to what is shown on Table 1, with 10 open and nine multiple choice questions.

**Table 1:** Questionnaire used in this survey.

1. Work setting (s): () hospital () medical office ()public clinic () other
2. What is your training level on hearing and its impairment (hearing loss)? () medical school () postgraduate () specific courses () other, which _____________________
3. Do you investigate your patients’ hearing? () yes () no. If so, at what age do you usually do it? () first 6 months() 2nd year () after 3 years () 1st year () 3rd year () other situations _____________
4. What procedure do you use___________________________
5. From what age do you understand it is possible to assess the hearing of a child? () First 6 months () 2º year() after 3 years () 1st year () 3rd year () other situations
6. Do you know about hearing assessment techniques in childhood? () yes () no
7. What is your approach when the mother complains about the child's hearing or speech delay? () carry out some test () refers to an audiologic test () refers to an otorhinolaryngologist () other __________________________________________________
8. What do you consider as criteria to refer a child to audiologic assessment? () when the mother asks for () when clinical evaluation suggests () when the child is under risk () routinely ()others ___________
9. Do you think early hearing impairment diagnosis is important? Why? () yes () no
10. In what ways do you think hearing deficiencies may impair the child's life?
11. What do you consider to be “high risk” for hearing?
12. Considering the child's auditory development, do you have any special approach in “high risk” cases? () yes() no If so, what is it? ___________
13. Regarding the previous question, if the answer is yes, at what age? () first 6 months() 2nd year () after 3 years () 1st year () 3rd year () other
14. If you work in a public health care facility, do you follow the child that has a hearing impairment? () yes () no
15. Resources are lacking (knowledge, equipment, etc.) for you to control children's hearing in public health care facilities? () yes() equipment () knowledge () other______________ () no
16. As of what age do you think a child may wear a hearing aid? () first 6 months () 2nd year() after 3 years () 1st year () 3rd year
17. Do you know the different types of hearing loss? () yes () no If so, how do you classify?______
18. Do you know the classifications as to the different levels of hearing loss? () yes () no If so, how do you use it?______________
19. It is believed that family and school play a fundamental role in the child's communication development, do you believe the physician may also play a role in it?

We sent out 46 questionnaires and 24 returned properly filled out. The average age of the interviewees was 44 years, averaging 21 years of license to practice medicine, 10 females and 14 males. It is worth highlighting that these data was not considered in the result analysis.

We started the research by interviewing each professional and explaining our intent. The interviewees decided to answer the questionnaire individually and later they returned it.

We have respected all research-related ethics, such as the interviewees’ identities. The present study has been approved by the Ethics committee of the University of Tuiuti - Paraná - UTP - and we obtained the authorization of the guardians through signing the informed consent form.

## RESULTS

We obtained 51% of answers. We did have some difficulties in carrying out this study, because the ambiguity of the answers has made it difficult to analyze the results. Since there were open answer questions, all the answers presented were organized.

Forty per cent of the pediatricians reported that they work in private offices, 31% in hospitals, 27% in public health care and 2% in other places.

As to their training in hearing impairment, 62% of the professionals said it happened during their undergraduate course, 21% through journals, 4% took specific courses and 13% did not answer it.

**Graph 1:**
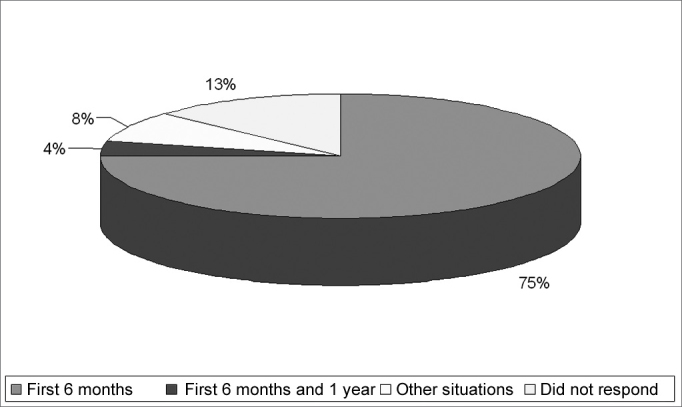
Age at which auditory investigation was performed.

Eighty-seven percent of the interviewed physicians said they check the hearing of their patients and 13% said they do not do it. Of these, 75% said it is possible to assess a child's hearing in the first six months of life, 4% from the first six months to one year, 8% in other situations and 13% did not answer (Graph 1).

Of the procedures used by those pediatricians who investigate the hearing of their patients, the methods used are the behavioral sound test, medical history, clinical examination, among others (Table 2).

**Table 2:** Procedures.

PROCEDURE	FREQUENCY
Perform behavioral sound tests	7
Medical interview	4
Clinical exam	4
Did not answer	4
Test the cochleo-eyelid reflex	2
Observe child's reactions	1
Refer to Otoacoustic Emissions Test	1
Assess the pychomotor development	1
Ask the parents to observe the baby	1
Refer to the otorhinolaryngologist	1
Refer to audiometry	1

As to the ideal age to assess hearing, 88% of the interviewees answered it was possible to be carried out in the first six months of life, 8% in other ages and 4% said it could only be done after three years of life. As to the techniques used in order to carry out hearing assessment, 83% said they knew about it but were not able to describe them, and 17% said they did not know any technique.

The approach followed by the pediatricians when the mother of their patients complained about their children's hearing or speech delay is to refer the patient to audiological evaluation (21%) or to an otorhinolaryngologist (38%), however, the total number of other procedures (41%) is rather significant.

Fifty percent of the pediatricians did not answer as to the criteria considered in order to perform the audiologic assessment, 42% reported a suggestive clinical assessment as a pre-requisite, 4% when the mother requests and 4% when the child is at risk.

The importance of hearing impairment early diagnosis proved to be evident in this question item, since there was unanimity (100%) in the answers.

The most frequent justifications for early diagnosis were: concern regarding early treatment, learning disorders prevention, speech development and child's stimulation (Table 3).

**Table 3:** Justification for early diagnosis in order of frequency.

IMPORTANCE	FREQUENCY
Treat early on	9
Prevent learning disorders	6
Help in neuropsychomotor development	4
Allow for proper speech development	3
Stimulate the child	3
Socialization	2
Facilitate rehabilitation (therapy)	1
Rehabilitate until seven years	1
Aid in language development	1
Preclude delays in overall development	1
Improve self esteem	1
Prevent more severe hearing problems	1
Treat before six months	1
Refer to hearing aid fitting	1
Refer to specific treatment	1

Among the major impairments caused by hearing loss, socialization was the most important one, learning difficulties, overall development delay, speech development delay and language development were also mentioned.

Considering high hearing impairment risk, prematurity was regarded as a major factor, followed by family history, congenital impairment and pregnancy specific diseases (Table 4).

**Table 4:** Factors considered as high risk for hearing.

HIGH RISK	FREQUENCY
Prematurity	12
Family history	10
Congenial impairment	8
Pregnancy-specific diseases	7
Did not answer	6
Use of ototoxic drugs	5
Repeated otitis media	3
Meningitis	3
Stay in ICU	2
Exposure to noise environments	2
Perinatal complications	2
Use of antibiotics	2
Traumas	1
Some diseases	1
Neurotrophic viruses	1
Small for gestational age	1
High risk pregnancy	1
Child that does not respond to stimulus at birth	1
Severe malnutrition	1
Low birth weight	1

The approach followed in high risk cases, for most of them (92%), is a referral to the otorhinolaryngologist, 4% referred to neonatal hearing screening and audiological assessment. Only 4% of the interviewees did not specify their answers. For high risk patients, 80% of the interviewees answered that they use a special approach in the first six months, 8% use other approaches, 8% did not answer and 4% did it in the first year of life.

Among physicians who work in public health care clinics, only 21% reported they follow up these hearing impaired children, 29% said they do not follow these children, and 50% did not answer.

**Graph 2:**
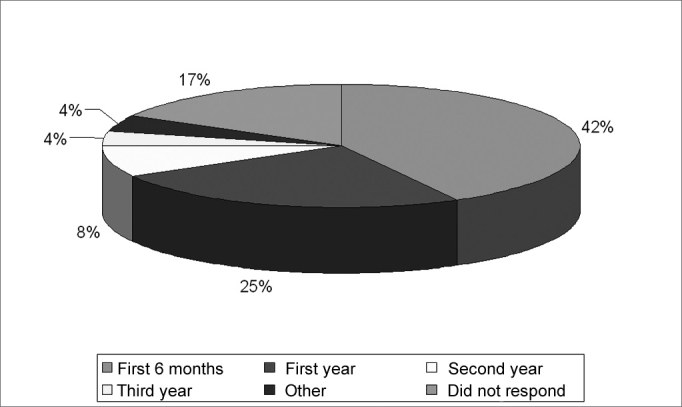
Age at which a child is already able to wear a hearing aid.

Thirty eight percent of the interviewees did not answer the question about available resources, 33% reported lack of equipment, 25% reported lack of resources and only 4% reported they had enough resources. Forty two percent of the interviewees answered that as of 6 months of age the child is able to wear a hearing aid, 25% said they could as of 1 year of age, 8% said second year, 4% third year, 4% above this age, and 17% did not answer (Graph 2).

Regarding hearing loss types, 45% of the interviewees said they were aware of those, 35% said they were not aware, and 20% did not answer. As to hearing loss levels, 71% of the pediatricians are not aware of the classifications, 25% said they were aware, and 4% did not answer.

There was an unanimous agreement as (100%) to the importance of the physician's role in the child's communication performance.

## DISCUSSION

The results found are similar to those found by Balieiro and Balieiro (1987)15; Weiss (1999)9; Bevilacqua et al.(2000)^16^ and Barros; Galindo & Jacob (2002)^14^.

The investigation carried out by Barros; Galindo & Jacob (2002)^14^ showed that pediatricians work in more than one place, and many of them acquired their knowledge on hearing impairment during their medical school years, agreeing to what we found in the present study.

Even when 75% of the physicians state that they do investigate the hearing status of their patients in their first six months of life and also said they are aware of the techniques used, we can see the difficulty they have to describe them. When we compare these findings with the ones from Balieiro and Balieiro (1987)^15^, Weiss (1999)^9^ and Barros; Galindo & Jacob (2002)^14^, we did not find significant differences.

Barros; Galindo & Jacob (2002)^14^ reported that 76% of the pediatricians believe it to be possible to carry out hearing assessment in the first six months of life, even if they do not do it themselves; 88% of the pediatricians interviewed in the present study also agree that hearing may be investigated at this age, but few of them actually do it.

When the mother complains of the child's hearing, Balieiro and Balieiro (1987)^15^ described that the procedure used by 50% of our interviewees is to confirm the complaints by their own means, and the remaining refer to the specialist. Again, there was a great similarity on the results obtained by this investigation, specially regarding referrals to the specialist; however, non-specific procedures and lack of response reached a significant number.

There was unanimity as to early hearing diagnosis, showing concern as to socialization and learning difficulties. This awareness about the importance of early diagnosis, as well as the losses caused by late diagnosis were also observed and reported by Bonamigo (1992)^18^.

Weiss (1999)^9^ described that the results justified the failure in establishing an early and effective hearing loss diagnosis, even when pediatricians and neonatologists are the target of many hearing-related complaints, since these complaints occur after two years of age, in their large majority. Now this same investigation has pointed out that there is relevant consideration as to clinical assessment and referrals to auditory investigation.

The present study findings have revealed the following as major risk factors: prematurity, family history and congenital hearing loss. The special approach was mentioned in these cases, and a referral to the specialist was a preponderant factor. Weiss^9^ (1999) mentioned that the otorhinolaryngologist is the chosen specialist in these situations. Balieiro and Balieiro (1987)^15^ showed the high number of professionals who did not know he risk factors affecting hearing.

Balieiro and Balieiro (1987)^15^ reported that among interviewees, most of them did not have any knowledge whatsoever about hearing screening and many totally ignored children hearing assessment methods. Barros; Galindo & Jacob (2002)^14^ mentioned that more than 70% of the interviewees stated it to be possible to routinely assess hearing in the first six month of life, however very few would do it. Rabelo et al. (2004)^17^ reported that 70.6% of the pediatricians order otoacoustic emissions and audiometry for newborns that use aminoglycosides. By comparing the findings of the aforementioned studies, we can see that pediatricians are now more aware of the importance of early auditory assessment. Even then, this study revealed a low number of physicians who refer patients to neonatal hearing screening.

The professionals who work in the public health network have complain about the lack of resources, equipment and specific knowledge to follow the development of hearing in children. Balieiro and Balieiro (1987)^15^ reported similar results, showing that over 60% of the pediatricians who work in the public heath care network did not carry out any sort of hearing investigation because of the same reasons.

There is a considerable number of professionals who say they believe a child may wear a hearing aid in his/her first year of life, however most of them agree that this may happen in their first six months of life, these results are in agreement with those found by Balieiro and Balieiro (1987)^15^.

Over 50% of the physicians surveyed by Barros, Galindo & Jacob (2002)^14^ and 45% of the ones interviewed in the present investigation responded positively as to their knowledge on the different types of hearing losses, however only one of them actually used the words: conductive, sensorineural and mixed.

In the study carried out by Barros, Galindo & Jacob (2002)^14^, as well as in the present one, we observed a low number of pediatricians with actual knowledge regarding the classification of the different degrees of hearing loss. The physician's concern regarding the child's communication was clear in the present investigation, matching the results found by Balieiro and Balieiro (1987)^15^.

## CONCLUSION

Even by dealing with a restricted sample, we concluded that since pediatricians are the professionals that have the closest contact with the child and his/her family, he/she is in charge of identifying as soon as possible those hearing impairment risks. The present investigation also made it very clear the need for early diagnosis and the methods used for that end; thus explaining the great importance of disclosing these data, having seen that the interviewees were genuinely interested in receiving such information.

## References

[1] Russo ICP, Santos TMM (1994). Audiologia Infantil.

[2] Marchiori LLM (2002). Análise das Alterações Auditivas em Escolares com Queixa de Problemas de Aprendizagem. Fono Atual.

[3] Schirmer CR, Fontoura DR, Nunes ML (2004). Distúrbios da aquisição da linguagem e da aprendizagem. J Pediatr.

[4] Cordero L, Chinski A, Breuning S, Sih T, Cinski A, Eavey R (2001). II Manual of Pediatric Otorhinolaryngology IAPO/IFOS. Copyright.

[5] Lewis DR, Costa Filho O.A., Campos CAH, Costa HOO (2003). Tratado de Otorrinolaringologia.

[6] Nóbrega M, Caldas N, Caldas SN, Sih T. (1999). Otologia e Audiologia em Pediatria.

[7] Jensen AMAR, Fragoso ACPF, Campos CAH, Costa HOO (2003). Tratado de Otorrinolaringologia.

[8] Finitzo T, Crumley WG (1999). Clínicas Pediátricas da América do Norte: Perda auditiva em crianças.

[9] Weiss KM (1999). Monografia de Conclusão do Curso de Especialização em Audiologia Clínica.

[10] Castro NP, Marone SAM, Almeida CIR, Redondo MC, Campos CAH, Costa HOO (2003). Tratado de Otorrinolaringologia.

[11] (2000). Comitê Brasileiro sobre Perdas Auditivas na Infância - Recomendação 01/99. J. Cons Fed Fonoaudiol.

[12] JOINT COMMITTEE ON INFANT HEARING - Year 2000 position statement (2000). Principles and guidelines for early hearing detection and intervention programs. Am J Audiol.

[13] Ramos BD, Caldas N, Caldas SN, Sih T. (1999). Otologia e Audiologia em Pediatria.

[14] Barros ACT, Galindo MC, Jacob RTS (2002). Conhecimento e conduta de pediatras frente à deficiência auditiva. Pediatr (São Paulo).

[15] Balieiro CR, Balieiro RO (1987). Diagnóstico da Deficiência Auditiva nos Primeiros Anos de Vida: Importância da Participação dos Pediatras. Revista Distúrbios da Comunicação.

[16] Bevilacqua MC, Bandini HH (2000). Tschiedel, Diagnóstico da Deficiência Auditiva na Infância: uma avaliação de nível de conhecimento dos pediatras de uma cidade do Centro Oeste Paulista. Pediatr Mod.

[17] Rabelo BGR, Salomão LM, Carnivali PA, Leite ICG (2004). Algumas considerações sobre o grau de conhecimento dos pediatras sobre questões fonoaudiológicas. Fono Atual.

[18] Bonamigo AW (1992). Audição na Infância: O conhecimento do Pediatra. Rev Distúrb Comun.

